# Positive association between mammographic breast density and bone mineral density in the Postmenopausal Estrogen/Progestin Interventions Study

**DOI:** 10.1186/bcr1327

**Published:** 2005-09-22

**Authors:** Carolyn Crandall, Shana Palla, Beth A Reboussin, Giske Ursin, Gail A Greendale

**Affiliations:** 1 Division of General Internal Medicine, David Geffen School of Medicine at University of California, Los Angeles, CA, USA; 2 Department of Public Health Sciences, Wake Forest University School of Medicine, Winston-Salem, NC, USA; 3 Department of Preventive Medicine, USC Norris Comprehensive Cancer Center, Los Angeles, CA, USA; 4 Department of Nutrition, University of Oslo, Oslo, Norway; 5 Department of Geriatrics, David Geffen School of Medicine at University of California, Los Angeles, CA, USA

## Abstract

**Introduction:**

Mammographic breast density is a strong independent risk factor for breast cancer. We hypothesized that demonstration of an association between mammographic breast density and bone mineral density (BMD) would suggest a unifying underlying mechanism influencing both breast density and BMD.

**Methods:**

In a cross-sectional analysis of baseline data from the Postmenopausal Estrogen/Progestin Interventions Study (PEPI), participants were aged 45 to 64 years and were at least 1 year postmenopausal. Mammographic breast density (percentage of the breast composed of dense tissue), the outcome, was assessed with a computer-assisted percentage-density method. BMD, the primary predictor, was measured with dual-energy X-ray absorptiometry. Women quitting menopausal hormone therapy to join PEPI were designated recent hormone users.

**Results:**

The mean age of the 594 women was 56 years. The average time since menopause was 5.6 years. After adjustment for age, body mass index, and cigarette smoking, in women who were not recent hormone users before trial enrollment (*n *= 415), mammographic density was positively associated with total hip (*P *= 0.04) and lumbar (*P *= 0.08) BMD. Mammographic density of recent hormone users (*n *= 171) was not significantly related to either total hip (*P *= 0.51) or lumbar (*P *= 0.44) BMD. In participants who were not recent hormone users, mammographic density was 4% greater in the highest quartile of total hip BMD than in the lowest. In participants who were not recent hormone users, mammographic density was 5% greater in the highest quartile of lumbar spine BMD than in the lowest.

**Conclusion:**

Mammographic density and BMD are positively associated in women who have not recently used postmenopausal hormones. A unifying biological mechanism may link mammographic density and BMD. Recent exogenous postmenopausal hormone use may obscure the association between mammographic density and BMD by having a persistent effect on breast tissue.

## Introduction

Mammographic density is a stronger risk factor for breast cancer than almost all other known breast cancer risk factors [1-5]. Bone mineral density (BMD) is another predictor of breast cancer risk in older women. Elderly women with high BMD have a greater risk of breast cancer than women with low BMD [6-10].

An underlying common pathway involving endogenous estrogen exposure or responsiveness to estrogen may link higher breast density, increased breast cancer risk, and greater BMD. Previous observations support this theory: markers of increased lifetime estrogen exposure are associated with higher breast cancer risk [11], higher BMD [12-14], and higher mammographic breast density [15,16]. However, it has not been established whether higher mammographic density is associated with higher BMD [17,18]. If mammographic density were associated with BMD, the notion of a common biological mechanism linking breast density, breast cancer risk, and BMD would be further supported.

To test whether BMD is an independent determinant of mammographic density in menopausal women, we examined baseline data from participants in the Postmenopausal Estrogen/Progestins Intervention Mammographic Density Study (PEPI-MDS), a substudy of the Postmenopausal Estrogen/Progestin Interventions Study (PEPI).

## Materials and methods

### PEPI

PEPI, designed to determine the effects of postmenopausal hormone therapy on cardiovascular risk factors and BMD in menopausal women, enrolled 875 menopausal women aged 45 to 64 years at seven clinical centers between 1989 and 1991. The eligibility criteria and study design have been reported in detail previously [19]. In brief, menopause was defined as the last menses having occurred at least 12 months ago. Women with any major contraindication for the use of estrogen or progestin treatment (for example breast cancer), or with any cancer other than basal cell skin cancer within the previous 5 years, were excluded. Additional exclusion criteria were the following: hysterectomy less than 2 months before screening, bilateral oophorectomy before age 44 years or more than 10 years previously, or serum levels of follicle-stimulating hormone less than 40 mIU/ml. Women who were using menopausal hormone therapy at the time of recruitment could join the trial if they stopped their privately prescribed hormone therapy at least 2 months before the first screening visit. For the present analysis, women who stopped their privately prescribed hormones to join PEPI are termed 'recent postmenopausal hormone users'.

Before recruitment, each clinic principal investigator obtained institutional review board approval for the informed consent information that was provided to study participants. All participants provided signed consent.

### PEPI-MDS

As part of the parent PEPI study, all enrolled women underwent mammography at baseline. PEPI-MDS, initiated after the parent PEPI, is intended to elucidate several hormonal, genetic, and lifestyle determinants of mammographic breast density in all participants in PEPI who had technically adequate mammograms.

### Measurements

We used baseline (before randomization) data from PEPI to determine the relationship between BMD and mammographic breast density. BMD was measured in duplicate with a Hologic QDR 1000 densitometer [20]. The *in vivo *coefficients of variation were 1.2% or less for measures of the hip and spine.

PEPI-MDS used mammograms obtained in the original PEPI to quantify mammographic density, the percentage of the breast composed of dense tissue, by using digitized mammograms, described in detail previously [21,22]. In brief, pre-randomization conventional craniocaudal mammograms performed between 1989 and 1994 during the original PEPI were retrieved from the seven participating PEPI clinical sites. Mammograms were scanned at a resolution of 150 pixels per inch with a Cobrascan CX-312T scanner (Radiographic Digital Imaging, Inc., Compton, CA) and Adobe Photoshop software (Adobe Systems, Inc., San Jose, CA) with ScanWizard 3.0.9, a specially designed program (Microtek International, Inc., Carson, CA). Breast tissue was outlined on a digitized mammogram image, and artefacts (for example pectoralis muscle) were excluded. Next, the reader established for each mammogram a 'yes/no' threshold above which breast tissue was defined as dense, and below which breast tissue was defined as not dense. Yellow tint was applied to areas of the image that were above this threshold; that is, dense. The software calculated percentage density as the ratio of the dense tinted area to the total area of the breast. The test–retest reliability for breast density, percentage breast density, and total breast area was high: intra-class correlation coefficients were more than 0.95 for mammograms that were rated not difficult to read or slightly difficult to read, and 0.91 to 0.95 for mammograms that were difficult or very difficult to read [22].

Covariates considered in this analysis were those related to mammographic density, BMD, or both [15,20,22-32], and include baseline, age, smoking history, use of postmenopausal hormone therapy before PEPI, and physical activity level (leisure, home, occupational), which were determined with standardized questionnaires [19]. Weight and height were measured, and body mass index (kg/m^2^) was calculated, at the screening and baseline visits, in accordance with a standardized algorithm [33].

### Statistical analysis

Generalized linear models were created with baseline percentage mammographic density as the outcome variable. Total hip BMD and baseline lumbar BMD were the two primary exposure variables. Covariates were chosen *a priori *on the basis of previous publications [15,20,22-32]. Models were adjusted for body mass index (continuous), age (continuous), and current cigarette smoking (yes/no). Because the relation between endogenous sex steroid hormones and mammographic density could be obscured by the effects of recent hormone use [22], analyses were stratified by whether women were recent hormone therapy users.

Although this study relies on the continuous BMD exposure variable for its primary analysis, we calculated adjusted mean percentage mammographic density in each quartile of lumbar spine BMD and total hip BMD as a means of illustrating the magnitude of the association between mammographic density and BMD among women who had not recently used postmenopausal hormones. All analyses were performed with Stata Version 7 (Stata Corporation, College Station, TX). All statistical tests were two-sided.

## Results

Of the 875 women in the original PEPI, 594 women had retrievable and technically adequate mammograms and thus comprised the PEPI-MDS sample. All 594 women in the PEPI-MDS underwent BMD measurement as part of the original PEPI. Because of missing covariate information, four women in PEPI-MDS were not included in the current analysis, leaving an analytic sample of 590 women. Characteristics of the PEPI mammographic density participants are displayed in Table 1, classified by previous menopausal hormone therapy use.

Mean age of the 590 women comprising the analytic sample was 56 years. On average, women had experienced menopause 5.6 years before enrollment in PEPI; 88% were Caucasian and few (13%) were current smokers. Recent users of postmenopausal hormones numbered 173 (29%); that is, they discontinued previously prescribed hormones to join PEPI. The mean duration since last use of hormone therapy at baseline was 4.1 months (range 3.1 to 5.1). Among the subset of women who stopped hormone therapy to join PEPI (that is, recent users), the mean duration since last use of hormone therapy was 6.5 months.

The multiple regression analyses in Table 2 show that, in women who were not recently exposed to postmenopausal hormone therapy, mammographic density was positively associated with both hip (*P *= 0.04) and lumbar (*P *= 0.08) BMD. In contrast, in the stratum of women who were recent postmenopausal hormone users, mammographic density was not significantly related to either hip (*P *= 0.51) or lumbar (*P *= 0.44) BMD.

**Figure 1:**
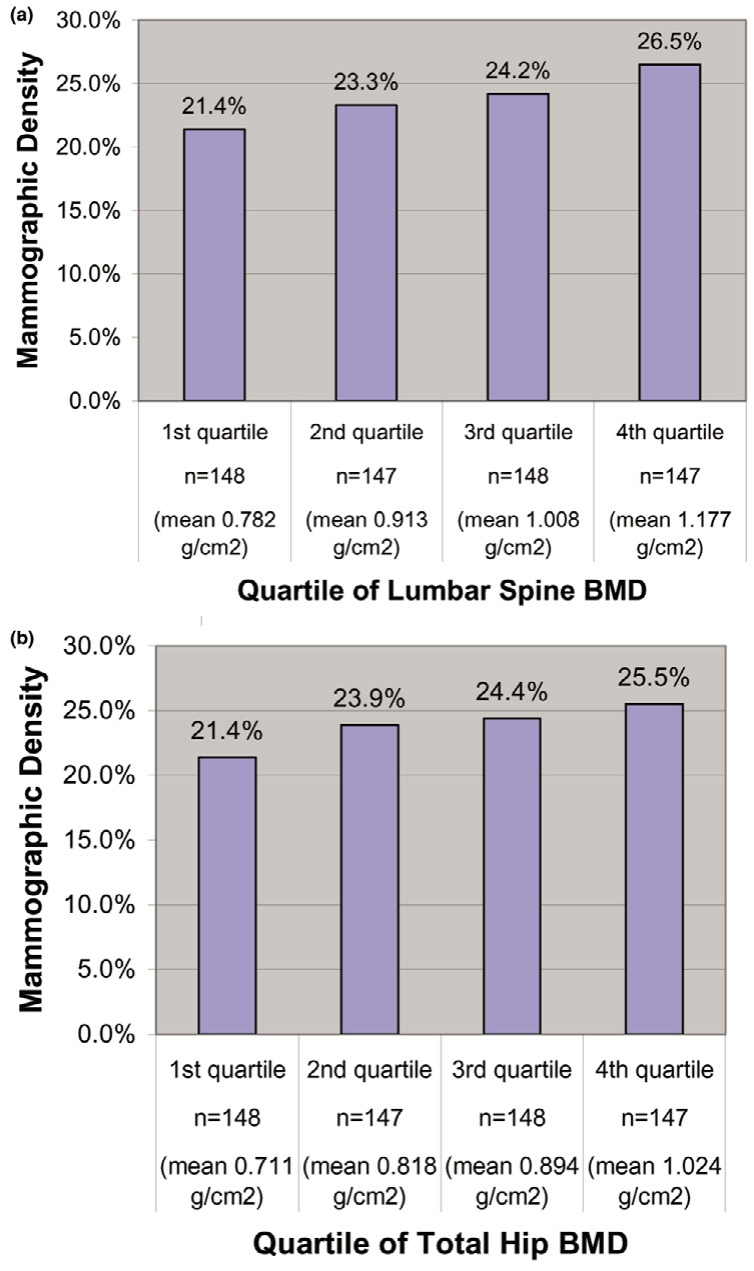
Association between mammographic density and bone density in women who were not recent hormone therapy users. (a) Mammographic density by quartile of lumbar spine bone mineral density (BMD) in  participants who were not recent hormone therapy users. Recent hormone users  were defined as women who stopped taking hormone therapy to join the  Postmenopausal Estrogen/Progestin Interventions Study (PEPI). Women who were  not recent hormone therapy users were women who did not stop taking hormone  therapy to join PEPI. **(b) **Mammographic density by quartile of total  hip BMD in participants who were not recent hormone therapy users.

To gauge the magnitude of the relation between percentage mammographic density and BMD, we calculated the adjusted mean percentage mammographic density by quartile of BMD (Figure 1). The increment in mammographic density between the lowest and the highest quartiles of spine BMD was 5% (Fig. 1a). The pattern of change in mammographic density across the quartiles of hip BMD (Fig. 1b) was similar to that of spine BMD, with an increment of 4% between the lowest and highest quartile of BMD.

## Discussion

In PEPI-MDS participants who had not recently used postmenopausal hormones, mammographic density was positively associated with total hip and lumbar spine BMD. By contrast, mammographic density was not associated with BMD in recent users of menopausal hormone therapy. Thus, exogenous postmenopausal hormone use (a mean of 6 to 7 months before baseline measurement) seems to obscure the association between mammographic breast density and BMD, presumably owing to a residual effect of recent hormone therapy use on breast density.

Findings of two previous publications are available for comparison with our findings. A cross-sectional population-based analysis of postmenopausal women found no significant association between BMD and breast density after adjustment for body mass index [17]. The previous study was not able to account for the timing of postmenopausal hormone therapy use in relation to mammographic density and BMD measurement, which had a major interactive effect in our study. Thus, the previous study's null finding might be due to a lack of stratification by current or previous hormone therapy use.

The second study found no relation between BMD and mammographic density after separately examining postmenopausal women using, and not using, hormone therapy [18]. However, that study assessed previous hormone therapy differently from our study in that they did not separately assess recent hormone users. Rather, the previous study classified recent users as nonusers. If, as our findings suggest, recent hormone use has residual effects that may obscure the association between mammographic density and BMD, the study's lack of separate analysis according to recent hormone use could be a reason why it did not find an association between mammographic density and BMD even in the nonusers. In addition, because the other study did not measure BMD in all women who had mammograms, it is possible that women who did receive BMD testing were different from those who did not receive BMD testing in certain key characteristics that obscured the association between mammographic density and BMD.

Despite the paucity of previously published studies directly examining a link between breast density and BMD, several lines of research are consistent with our findings: BMD is associated with breast cancer risk [6,7] and with indicators of endogenous estrogen exposure [13,34], and mammographic breast density is also associated with breast cancer risk [1-4] and with indicators of exposure to endogenous estrogen [15,16,35].

In addition to biological plausibility as described above, our results are congruent with the known effects of pharmacologic agents, such as hormone therapy or estrogen antagonists. For example, in the original PEPI, administration of combination estrogen–progestin hormone therapy to menopausal women increased BMD by between 2% and 5% over 36 months [20] and also increased mammographic breast density by between 3% and 5% over 12 months [22,36]. The degree of increase in estrone level [37,38] and estradiol level [38] predicts the increase in mammographic density with administration of exogenous estrogen and progestin hormone.

Although this study was not designed to measure hormonal markers, we can postulate that other hormonal factors besides estrogen may jointly affect BMD and mammographic density and explain their association in this analysis. Evidence from a randomized trial would arouse suspicion that progesterone might be an important mediator of mammographic density. PEPI found that mammographic density was increased only with the administration of combined estrogen and progestin, not estrogen alone, compared with placebo [22]. Although one study found progesterone level to be unrelated to mammographic breast density [39], and one study found no association between endogenous progesterone levels and BMD [40], a possible role for endogenous progesterone in determining both mammographic density and BMD warrants investigation. Alternatively, the mechanism underlying the association between mammographic density and BMD might involve other steroid sex hormones.

Whether moderated by estrogen alone or with other factors, the association we report between BMD and mammographic breast density suggests that there is a unifying biological mechanism behind BMD, mammographic density, and breast cancer risk. Moreover, we witnessed a 4 to 5% difference in mammographic density in the highest compared with the lowest quartile of BMD. This suggests an effect of biological consequence: relative risk of breast cancer increases by between 1.5% and 2% for each 1% increment in endogenous breast density [4,5]. Breast density is a strong risk factor for breast cancer, and its determinants are currently poorly understood. Linking breast density with other better-known biological outcomes, such as BMD, may be a step towards our understanding of what determines this strong risk factor for breast cancer.

Strengths of our study include large sample size, the ability to control for several potential confounders, contemporaneous ascertainment of mammograms and bone density tests on all participants, and detailed information about previous postmenopausal hormone exposure. However, our cross-sectional study design does not permit attribution of causation. The study design did not allow us to determine the association between BMD or breast density and breast cancer. It is unknown whether our findings apply to women in other age groups (elderly, premenopausal) or ethnic groups (our population was 70% Caucasian).

## Conclusion

In conclusion, mammographic density was not associated with BMD in recent users of postmenopausal hormone therapy, which is consistent with a residual effect of hormone therapy on mammographic density. In women who were not recent users of postmenopausal hormones, mammographic density was positively associated with BMD, suggesting a unifying biological mechanism linking BMD and mammographic density.

## Abbreviations

BMD = bone mineral density; PEPI-MDS = Postmenopausal Estrogen/Progestin Interventions Mammographic Density Study; PEPI = Postmenopausal Estrogen/Progestin Interventions Study.

## Competing interests

The authors declare that they have no competing interests.

## Authors' contributions

CC and GG participated in the design of the analysis and drafted the manuscript. UG performed the mammographic density assessments. SP and BR performed the statistical analyses. All authors read and approved the final manuscript.

## Figures and Tables

**Table 1:** Demographic and behavioral characteristics of PEPI-MDS participants by recent use of menopausal hormones

Characteristic	Quit hormones to join PEPI (*n *= 173)^a^			Did not quit hormones to join PEPI (*n *= 143)			Never used hormone therapy (*n *= 274)^b^		
	Mean (SD)	Frequency (%)	*P*^c^	Mean (SD)	Frequency (%)	*P*^d^	Mean (SD)	Frequency (%)	*P*^e^
Age, years	55.9 (4.1)		0.27	56.4 (4.8)		0.31	55.9 (4.2)		0.84
Years since menopause	5.6 (2.5)		0.26	6.0 (2.6)		0.15	5.5 (2.8)		0.86
Body mass index, kg/m^2^	25.2 (4.1)		0.003	26.7 (4.5)		0.66	26.5 (4.7)		0.002
Months since menopausal hormone use^f^	6.5 (12.0)		<0.001	47.8 (60.0)		N/A	N/A		N/A
Alcohol use, g/day	6.2 (9.9)		0.99	6.1 (12.1)		0.67	6.7 (14.1)		0.66
Percentage mammographic density	0.28 (0.18)		0.01	0.23 (0.17)		0.68	0.23 (0.18)		0.01
Total hip bone mineral density, g/cm^2^	0.866 (0.128)		0.82	0.863 (0.127)		0.71	0.858 (0.120)		0.51
Lumbar bone mineral density, g/cm^2^	0.997 (0.164)		0.01	0.952 (0.154)		0.54	0.962 (0.154)		0.02
Non-white, number (%)		13 (7.5)	0.007		25 (17.5)	0.10		32 (11.7)	0.15
Smoking									
Current		10 (5.8)	0.01		24 (16.1)	0.74		43 (15.7)	0.006
Former		73 (42.2)			55 (38.5)			96 (35.0)	
Never		90 (52.0)			65 (45.5)			135 (49.3)	
Physical activity level, number (%)^g^									
Low (score < 1.5)		47 (27.1)	0.04		58 (40.6)	0.31		91 (33.2)	0.40
Medium (score = 1.5)		64 (37.0)			42 (29.4)			95 (34.7)	
High (score > 1.5)		62 (35.8)			43 (30.1)			88 (32.1)	

Of the 594 women who had adequate mammographic density measurements, 4 did not have complete information on other variables and are not included in the analyses. The table displays information regarding the remaining 590 women.

^a^Women who were using menopausal hormone therapy at the time of recruitment could join the trial if they stopped their privately prescribed hormone therapy at least 2 months before the first screening visit. A total of 317 participants answered the question regarding whether they quit menopausal hormone therapy to join PEPI.

^b^This column also includes women who did not remember whether they had used hormone therapy.

^c^*P *value comparing the characteristic in the participants who quit hormones to join PEPI with the participants who did not quit hormones to join PEPI.

^d^*P *value comparing the characteristic in the participants who did not quit hormones to join PEPI with the participants who never used hormone therapy.

^e^*P *value comparing the characteristic in the participants who never used hormone therapy with the participants who quit hormones to join PEPI.

^f^Number of months since menopausal hormone therapy use in women who had used hormones previously. In the overall sample, the mean duration since last use of hormone therapy was 4.1 months (range 3.1 to 5.1 months).

^g^Leisure and home physical activity self-rated by participants: inactive (1), light (2), moderate (3).

PEPI, Postmenopausal Estrogen/Progestin Interventions Study; PEPI-MDS, PEPI Mammographic Density Study.

**Table 2:** Association between mammographic breast density and bone mineral density stratified by recent hormone therapy use

Sample	Total hip BMD (g/cm^2^)		Lumbar spine BMD (g/cm^2^)	
	β, mean (SEM)	*P*	β, mean (SEM)	*P*
Not recent hormone users (*n *= 417)	0.156 (0.076)	0.04	0.099 (0.056)	0.08
Recent hormone users (*n *= 173)	-0.073 (0.110)	0.51	-0.065 (0.083)	0.44

Mammographic density was expressed as the percentage of the breast composed of dense tissue by using a semi-quantitative method [21]. The outcome of linear regression models was percentage mammographic density. Models were adjusted for body mass index (kg/m^2^, continuous), age (continuous), and current smoker (versus former or never smoker). Recent hormone users were defined as women who had stopped taking hormone therapy for PEPI.

BMD, bone mineral density; PEPI, Postmenopausal Estrogen/Progestin Interventions Study.
